# Efficacy of high-frequency rTMS in the treatment of gait disorder and cognition in patients with Parkinson’s disease based on wearable devices and eye-movement assessments

**DOI:** 10.3389/fnagi.2024.1480171

**Published:** 2024-10-16

**Authors:** Hong Yin Tang, XiangLian Liao, Peng Li, Pengfei Zhang, Jian Yao, Yilan Xing, Xin Zhao, Xuying He, Jie Zan, Guihua Li

**Affiliations:** ^1^Department of Neurology, The Affiliated Guangdong Second Provincial General Hospital of Jinan University, Guangzhou, China; ^2^The Third People’s Hospital of Baiyun District, Guangzhou, Guangdong, China; ^3^The Second School of Clinical Medicine, Southern Medical University, Guangzhou, Guangdong, China; ^4^Department of Anesthesiology and Surgery, Liwan Central Hospital of Guangzhou, Guangzhou, China; ^5^Guangdong Key Laboratory of Nanomedicine, CAS-HK Joint Lab of Biomaterials, CAS Key Laboratory of Biomedical Imaging Science and System, Chinese Academy of Sciences, Shenzhen, China; ^6^School of Biomedical and Pharmaceutical Sciences, Guangdong University of Technology, Guangzhou, Guangdong, China

**Keywords:** Parkinson’s disease, wearable gait analyzer, eye movement assessments, gait disorder, cognition

## Abstract

**Background:**

Postural instability and gait disorder and cognitive dysfunction are common symptoms of Parkinson’s disease (PD). Scale assessment is frequently used in the clinic to evaluate PD, but this technique is limited by its lack of sensitivity to changes in disease progression and its difficulty in capturing subtle movements and changes in cognitive function. It is currently believed that high-frequency repetitive transcranial magnetic stimulation (rTMS) can improve motor and cognitive dysfunction in patients with PD, though it remains controversial. Therefore, it is imperative to monitor and dynamically identify changes in postural instability and gait disorder, as well as those in cognitive dysfunction, in PD to develop targeted interventions. In this study, we observed the effect of high-frequency rTMS on gait disorders and cognitive functions in patients with PD by comparing data from wearable devices and eye-tracking devices before and after treatment.

**Methods:**

A total of 159 patients with PD were included in this study. A GYENNO MATRIX wearable gait analyzer was used to monitor the objective gait data (including the timed up-and-go, narrow-track, and turning tests), the Eyeknow eye-tracking evaluation system was used to monitor the patient’s eye movement cognition data (including the smooth pursuit, pro-saccade, and anti-saccade tests), and gait and cognitive function–related scales, including the Tinetti Balance Scale, Tinetti Gait Scale, Berg Balance Scale, Mini-Mental State Examination, and Montreal Cognitive Assessment (MoCA), were evaluated at the same time before and after high-frequency rTMS treatment.

**Results:**

The mean step length, mean stride velocity, stride length, and mean step frequency of patients with PD in the timed up-and-go test all increased compared with those before rTMS treatment, whereas the mean stride time and double support decreased. In the narrow-track test, the mean stride velocity increased and the mean stride time decreased. In the turning test, the turning left duration, turning right duration, mean duration, mean number of steps, and average step duration decreased, while the mean angular velocity increased after rTMS treatment. Compared with those before rTMS treatment, the latency period of patients with PD in overlapping saccades decreased, the completion time of overlapping saccades decreased, and the average saccade speed increased. In the anti-saccade test, the completion time decreased and the average saccade speed increased after rTMS treatment. Compared with those before rTMS treatment, the Tinetti Balance Scale, Tinetti Gait Scale, Berg Balance Scale, Mini-Mental State Examination, and MoCA scores increased, and the MoCA sub-items improved in terms of visual–spatial and executive function, language, abstraction, delayed recall, and orientation after rTMS treatment.

**Conclusion:**

High-frequency rTMS may be an effective therapy for improving gait disorders and cognitive functions in patients with PD.

## 1 Introduction

Postural instability and gait disorder (PIGD) and cognitive dysfunction are common motor and non-motor symptoms of Parkinson’s disease (PD), respectively (Jankovic, 2008; Aarsland et al., 2017). Based on the Hoehn and Yahr (H-Y) staging system, postural instability and gait disorder can be divided into early, middle, and late stages (Fang et al., 2020). In its early stages, patients may have increased gait variability, such as a slower gait speed and smaller stride length (Wu et al., 2015). In its middle stages, patients may have symptoms in both limbs, alongside more sluggish movements, increased support in both lower limbs, and a further reduced arm swing (Wu et al., 2015). Moreover, abnormal changes in posture (such as leaning forward) may interfere with gait kinematics, which can lead to the further aggravation of gait abnormalities, such as freezing of gait and festination. Middle- and late-stage gait disorders increase the risk of falls, fractures, and even death (Mirelman et al., 2019). In addition, cognitive dysfunction can occur at any stage of PD, and there is a dearth of effective biomarkers for assessing the degree of cognitive decline and predicting the progression of the disease, which can gradually lead to dementia and the inability to perform daily life activities. Therefore, the dynamic monitoring and treatment of gait disorders and cognitive dysfunction is a challenging problem that must be solved.

Scale assessments are frequently used in the clinic to evaluate gait disorders in patients with PD; however, some of them have limited validity and reliability (Ebersbach et al., 2006). Some studies (Cao et al., 2020; Zhan et al., 2018) have confirmed that wearable devices have high specificity and sensitivity for the early diagnosis and differential diagnosis of PD, and they can be used to quantify various gait characteristics (including speed, variability, and asymmetry). In recent years, it has been found that eye tracking, a nonverbal technique, is a less cognitively demanding method for measuring disease progression in patients with cognitive impairment (Wilcockson et al., 2019; Tao et al., 2020). Eye tracking also has a good correlation with traditional cognitive assessment scales (Polden and Crawford, 2023), suggesting that eye tracking can be used to assess and monitor the cognitive status, disease severity, and disease progression of patients with PD. The worsening of visually guided saccades is correlated with the severity of cognitive decline (Macaskill et al., 2012).

In recent years, an increasing number of studies (Khedr et al., 2006; Makkos et al., 2016; Jiang et al., 2020) have shown that repetitive transcranial magnetic stimulation (rTMS) has a significant effect on motor symptoms, as well as on some non-motor symptoms, in patients with PD. High-frequency rTMS is effective in improving the motor symptoms of PD, particularly in the bilateral motor cortex (Lefaucheur et al., 2020), and high-frequency (5 Hz or higher) stimulation of the primary motor cortex significantly improves the motor symptoms associated with PD (Zanjani et al., 2015). However, the efficacy of rTMS in the treatment of non-motor symptoms in patients with PD remains controversial (Makkos et al., 2016; Chen, 2018). The origin of this controversy lies in the fact that the selection of treatment parameters (such as frequency, target, treatment duration, and treatment course) is not fixed, and it is therefore inconclusive whether different targets and frequencies are required for different symptoms. Furthermore, Goldsworthy et al. (2021) highlighted that the relationship between pre-stimulation neural variability and subsequent rTMS-induced neuroplasticity deserves further exploration.

Therefore, in this study, we sought to better evaluate the gait and cognitive function of patients with PD and directly observe the efficacy of rTMS treatment. Consequently, we conducted a clinical evaluation survey and assessment based on wearable device data and the eye movements of patients with PD who were hospitalized at the neurology clinic of the Second People’s Hospital of Guangdong Province between January 2020 and August 2023. We analyzed changes in the gait and saccade parameters of patients with PD before and after high-frequency rTMS treatment to further evaluate the effect of high-frequency rTMS on motor and cognitive functions in patients with PD. Our preliminary findings provide a new method for exploring the monitoring and treatment of gait and cognitive impairment in patients with PD, provide an objective basis for the formulation of treatment plans for PD gait and cognitive dysfunction, and accelerate clinical translation.

## 2 Participants and methods

### 2.1 Subjects

A prospective observational research study was conducted in the departments of the Second People’s Hospital of Guangdong Province from January 2020 to September 2023. A total of 159 PD patients met the criteria for idiopathic PD diagnosed according to the UK Brain Bank criteria, and their inclusion was confirmed by two senior physicians.

#### 2.1.1 Inclusion and exclusion criteria

The inclusion criteria were as follows: (1) patients met the criteria for idiopathic PD diagnosed according to the UK Brain Bank criteria as confirmed by two senior physicians; and (2) patients completed all survey scales and provided basic clinical data.

The exclusion criteria were as follows: (1) patients had other types of parkinsonism, including secondary parkinsonism, parkinsonism superimposed syndrome, or familial parkinsonism; (2) patients had other diseases with gait disturbance (such as spinal joint injury, muscle spasm, stroke, peripheral neuropathy, muscular diseases, hydrocephalus, or cognitive impairment); had experienced organ failure (such as of the heart, lung, liver, or kidney); had a malignant tumor, unstable condition, or serious internal disease; or exhibited severe, psychotic, or uncooperative behavior; (3) patients had neuropsychiatric diseases (such as schizophrenia, severe anxiety, or depression); (4) patients had cognitive impairment caused by stroke, brain tumor, hydrocephalus, or another cause; (5) patients had undergone deep brain stimulation; or (6) patients were unable to complete the scale and eye movement examination because of poor hearing, poor vision, hand function disability, or another factor.

### 2.2 Clinical assessment and groups

The trial was reviewed and approved by the Ethics Committee of the Second People’s Hospital of Guangdong Province. All participants provided their written informed consent. All participants completed a survey to collect epidemiological data, including basic demographics and relevant clinical assessments. The survey included the following information: name, gender (male/female), age (years), age at onset (years), disease duration (years), mode of onset, marital status, educational level, use of anti-Parkinson drugs, and other general information.

### 2.3 Eyeknow wearable sensor

**Figure 1:**
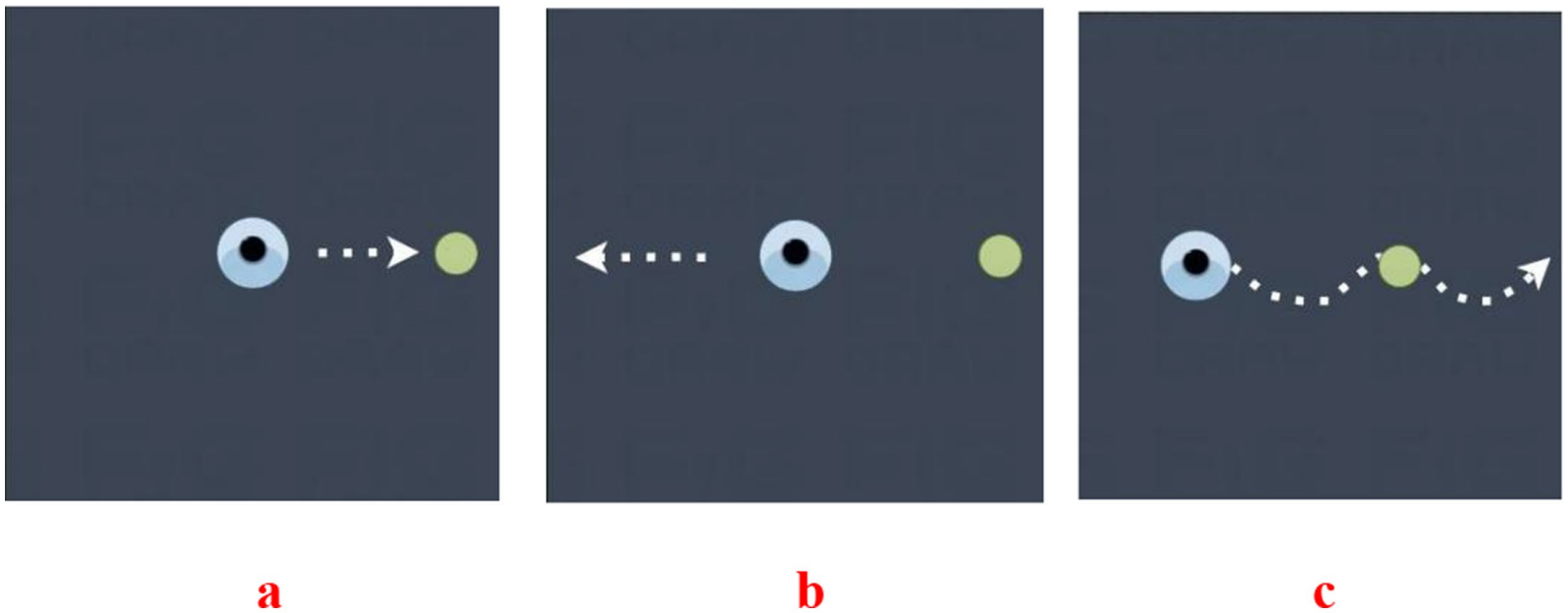
Diagram of the Eyeknow. **(a)** Reflexive vision-guided scanning diagram: (1) the subject looks at the target point in the middle of the screen; (2) the target point appears randomly around the screen; and (3) the subject quickly looks at the random target point. **(b)** Anti- saccade diagram: (1) the subject looks at the target point that appears in the center of the screen; (2) the central target point disappears and a random target point appears around the screen; and (3) the subject looks in the opposite direction to the target point. **(c)** Smooth tracking diagram: the subjects looked at the target point and followed the target point to move at the same speed in the same direction until the target point disappeared.

The Eyeknow is a wearable sensor used to monitor eye movements (Li et al., 2024; Weng et al., 2023; Lin et al., 2024). It uses infrared eye movement capture technology combined with computer vision to digitally reproduce the trajectories of the spatiotemporal activity of the eyes. Based on immersive eye movement guidance technology in virtual reality, the Eyeknow effectively ensures the subjects’ concentration, allowing them to perform classic eye movement tests while the spatiotemporal trajectories of their eye movements are digitally recorded. Through a spatiotemporal sequence data analysis model, the eye movement trajectories and key motion parameters are efficiently calculated and analyzed, and the subjects’ eye movements are quantitatively analyzed and evaluated based on objective data. Using the eye-tracking evaluation system, we recorded eye movements at a sampling rate of 120 Hz. Before the experiment, eye movements were calibrated using a 9-point calibration procedure to ensure that the error of the acquisition was no more than 2° (i.e., the target point must fall within a circle of visual angle with a radius of 2°). The assessment consisted of three tasks: overlapping saccades (reflex saccades), anti-saccades, and smooth pursuit. The participants performed saccades in or away from the direction of the stimulus or moved their eyes to the target point according to the task instructions. The participants performed 16 trials in each task, with a target point occurrence interval of 2 s and a target point angle of 15° (Figure 1).

### 2.4 GYENNO MATRIX wearable device

**Figure 2:**
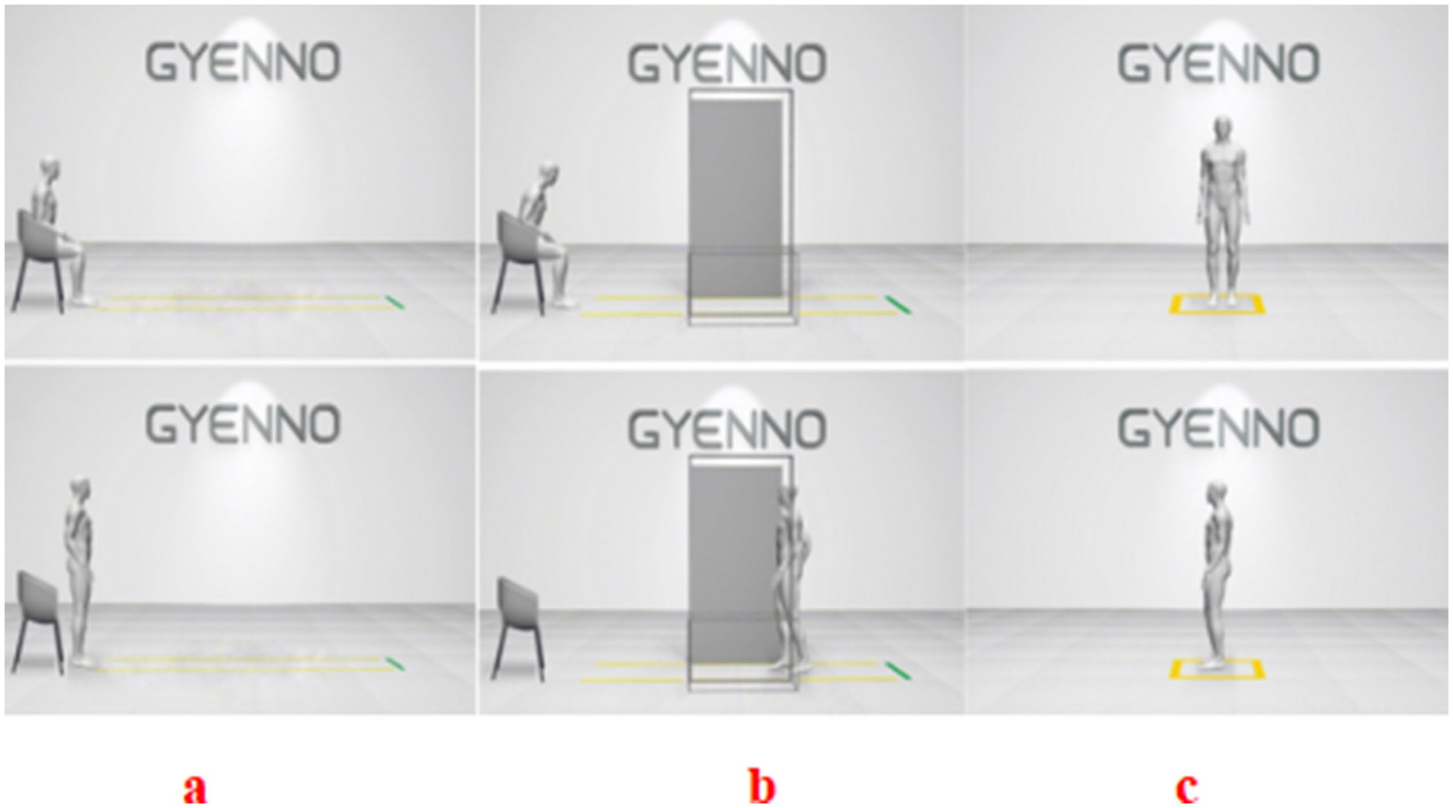
Three assessments using the GYENNO MATRIX wearable device. **(a)** Timed up-and-go (TUG) test: the patients were instructed to stand up from a chair, walk in a straight line for 5 m at a comfortable speed, take a 180° turn at the 5-m marker, walk back to the starting point, take a 180° turn in front of the chair, and then sit back down on the chair. **(b)** Narrow-track test: the patients were instructed to pass through a narrow passage that was two fists wider than they were and walk in a straight line for 5 m at a comfortable speed. **(c)** Turning test: the patients were instructed to turn in a circle twice to the left and twice to the right.

A commercially available wearable motion and gait quantification assessment system, GYENNO MATRIX (Gyenno Science, Shenzhen, China), was used in this study. The device was approved by the National Medical Products Administration, the U.S. Food and Drug Administration, and the Conformité Europëenne Medical. The kinematic and dynamic parameters of human movement were collected by sensor devices placed at 10 data nodes: the chest, waist, left and right wrists, left and right thighs, left and right lower legs, and left and right feet. The measured parameters were transmitted to the operation center in real time using wireless transmission technology for three-dimensional movement postural reconstruction. Based on these data, the gait, postural balance, arm swing, and whole-body movement coordination of the patients were assessed. During the timed up-and-go test, the patients were instructed to stand up from a chair, walk in a straight line for 5 m at a comfortable speed, take a 180° turn at the 5-m marker, walk back to the starting point, take a 180° turn in front of the chair, and then sit back down on the chair. During the narrow-track test, the patients were instructed to pass through a narrow passage that was two fists wider than they were and walk in a straight line for 5 m at a comfortable speed. During the turning test, the patients were instructed to turn in a circle twice to the left and twice to the right. The patients were tested at their usual normal pace in all of the tests. The patients were tested during OFF medication because there were more serious gait disorders (Figure 2).

### 2.5 Gait-related and other scales

The gait-related and other scales employed in this research included the MDS Unified Parkinson’s Disease Rating Scale III component assessments, H-Y stage, Tinetti Balance Scale, Tinetti Gait Scale, Berg Balance Scale, Mini-Mental State Examination (MMSE), and Montreal Cognitive Assessment (MoCA). The MoCA is divided into eight sub-items: visual–spatial, executive function, naming, attention, language, abstraction, delayed recall, and orientation. These scales were used to evaluate patients both before and after the high-frequency rTMS treatment.

### 2.6 Repetitive transcranial magnetic stimulation

The method used for rTMS (carried out using a device manufactured by Beijing Gaosi Mingchuang Science and Technology Co., Ltd.) was as follows. Patients were first instructed to sit on the treatment chair. The coil center right against their pterion 1.5 cm above was stimulated by an 8-shaped coil probe (70 mm in diameter). The target of stimulation was located at the dorsolateral prefrontal cortex of the left frontal lobe (the F3 point according to the international 10/20 system), which is where Parkinson’s disease is typically treated. The stimulation frequency used in the treatment was 25 Hz, and the coil position was fixed during the magnetic stimulation. Each participant received a total of 1,000 stimuli at 90% of the motor threshold. The treatment continued for 10 days, with 20 min of treatment each day. Three assessments were recorded before and after the 10-day treatment of rTMS (25 Hz).

### 2.7 Statistical analysis

Paired t tests with SPSS 25.0 software were used for statistical analysis. The measurement data were presented as the mean ± standard deviation, and the count data were presented as a ratio. The χ^2^ (chi-square test) was used for categorical variables, and *p* < 0.05 was considered to indicate statistical significance.

### 2.8 Flowchart

**Figure 3:**
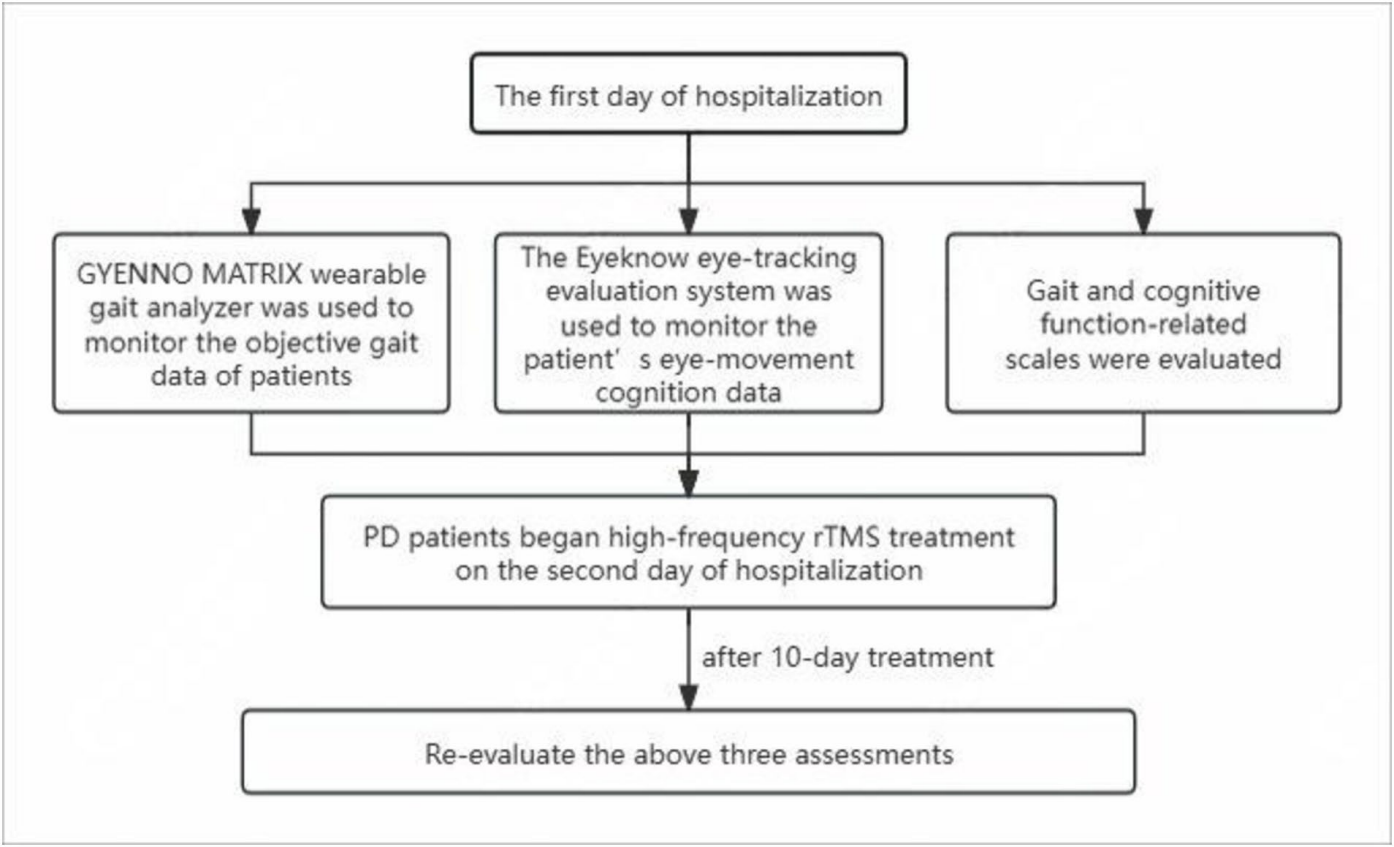
Flowchart of the study design.

The scales and use of the GYENNO MATRIX and Eyeknow eye-tracking systems were evaluated on the first day of hospitalization. On the second day, PD patients started high-frequency rTMS treatment for 10 days. The day after the completion of treatment, all PD patients were re-evaluated using the aforementioned three assessments. Figure 3 shows the flowchart of the study.

## 3 Results

### 3.1 General information on patients with PD

We recruited a total of 159 patients (93 men and 66 women) with PD as the case group who had an average age of 66.64 ± 11.09 years, a mean H-Y stage of 2.23 ± 1.15, a mean disease duration of 4.12 ± 2.61 years, and an equivalent daily dose of Madopar (200 mg levodopa and 50 mg benserazide) of 475.00 ± 19.43 mg. According to the MMSE score, 100 patients had cognitive dysfunction and 59 did not (Table 1).

**Table 1:** Baseline clinical characteristics of the PD patients.

Characteristics	PD patients
Sex (male/female)	93/66
Age (years)	66.64 ± 11.09
Disease duration (years)	4.12 ± 2.61
H-Y stage	2.23 ± 1.15
Cognitive dysfunction (Y/N)*	100/59
Gait disorder (Y/N)	73/86

*Y, yes; N, no. PD, Parkinson’s disease; H-Y, Hoehn and Yahr.

### 3.2 Comparison of gait parameters of wearable devices before and after treatment

Compared with those before rTMS treatment, the mean step length, mean stride velocity, stride length, and mean step frequency of patients with PD in the timed up-and-go test all increased after rTMS treatment, whereas the mean stride time and double support decreased. In the narrow-track test, the mean stride velocity increased and the mean stride time decreased after rTMS treatment. In the turning test, the turning left duration, turning right duration, mean duration, mean number of steps, and average step duration decreased, while the mean angular velocity increased, after rTMS treatment (Table 2).

**Table 2:** Comparison of wearable device parameters before and after rTMS treatment.

	Characteristics	Before treatment	After treatment	*t*	*p*
Timed up-and-go test	Mean step length (cm)	38.98 ± 10.60	41.75 ± 10.55	−4.707	<0.001*
	Mean stride velocity (m/s)	0.66 ± 0.23	0.73 ± 0.21	−4.698	<0.001*
	Stride length (cm)	76.95 ± 21.00	81.52 ± 20.72	−3.622	0.001*
	Mean step frequency (step/min)	101.53 ± 12.12	105.17 ± 12.25	−3.001	0.004*
	Mean stride time (sec)	1.21 ± 0.15	1.14 ± 0.15	3.339	0.002*
	Double support (%)	0.24 ± 0.07	0.22 ± 0.06	4.475	<0.001*
Narrow-track test	Mean step length (cm)	36.71 ± 11.75	37.41 ± 10.88	−0.963	0.340
	Mean stride velocity (m/s)	0.63 ± 0.24	0.67 ± 0.22	−2.096	0.041*
	Stride length (cm)	73.58 ± 23.71	74.79 ± 22.04	−0.872	0.387
	Mean step frequency (step/min)	101.91 ± 12.03	105.52 ± 11.97	−1.396	0.168
	Mean stride time (sec)	1.19 ± 0.14	1.12 ± 0.15	4.158	<0.001*
	Double support (%)	0.24 ± 0.08	0.23 ± 0.07	1.918	0.060
Turning test	Turning left duration (sec)	25.55 ± 31.77	22.21 ± 30.99	2.832	0.006*
	Turning right duration (sec)	26.64 ± 40.98	21.39 ± 35.48	2.207	0.032*
	Mean duration (sec)	26.40 ± 35.63	21.82 ± 32.93	2.816	0.007*
	Mean number of steps (step)	32.92 ± 27.39	27.96 ± 22.51	2.560	0.013*
	Mean angular velocity (m/s)	47.77 ± 27.82	52.96 ± 26.71	−2.603	0.012*
	Average step duration (sec)	0.63 ± 0.18	0.58 ± 0.15	3.465	0.001*

*p < 0.05; *p*-values are the result of paired t and *χ*^2^ tests. rTMS, repetitive transcranial magnetic stimulation.

### 3.3 Comparison of eye-movement parameters before and after rTMS

Compared with those before rTMS treatment, the latency period of patients with PD in overlapping saccades decreased after rTMS treatment, the completion time of overlapping saccades decreased, and the average saccade speed increased after rTMS treatment. In the anti-saccade test, the completion time decreased and the average saccade speed increased after rTMS treatment (Table 3).

**Table 3:** Comparison of eye-movement parameters before and after rTMS treatment.

	Characteristics	Before treatment	After treatment	*t*	*p*
Pro-saccade	Correctness (%)	94.50 ± 14.94	95.89 ± 10.58	−0.577	0.566
	Latency period (ms)	381.01 ± 127.93	330.59 ± 63.43	2.677	0.01^*^
	Completion time (average, ms)	452.43 ± 166.91	398.98 ± 100.38	2.061	0.044^*^
	Average saccade speed (°/s)	200.61 ± 77.28	254.98 ± 98.77	−2.915	0.005^*^
Anti-saccade	Correctness (%)	47.32 ± 28.23	42.37 ± 35.98	0.774	0.442
	Completion time (average, ms)	447.61 ± 109.77	404.74 ± 96.16	2.067	0.044^*^
	Error correction rate (%)	73.49 ± 33.97	82.01 ± 29.19	−1.219	0.229
	Latency period (ms)	362.62 ± 95.03	333.67 ± 90.78	1.478	0.145
	Duration of correction (average, ms)	369.80 ± 115.24	345.58 ± 128.75	0.99	0.328
	Average saccade speed (°/s)	205.60 ± 66.62	245.86 ± 81.65	−2.72	0.009^*^
Smooth pursuit	Startup duration (ms)	1,266.97 ± 3,212.41	1,180.95 ± 2,970.55	0.141	0.889
	Track speed (°/s)	31.28 ± 42.42	19.76 ± 10.99	1.877	0.066
	Number of offsets (time)	41.19 ± 40.09	54.74 ± 50.32	−1.549	0.127
	Total offset (>4°, °)	216.92 ± 217.21	313.12 ± 316.96	−1.831	0.073

^*^p < 0.05; *p*-values are the result of paired t and *χ*^2^ tests. rTMS, repetitive transcranial magnetic stimulation.

### 3.4 Comparison of relevant scale scores before and after rTMS treatment

Compared with those before rTMS treatment, the Tinetti Balance Scale, Tinetti Gait Scale, and Berg Balance Scale scores increased; after rTMS treatment, the MMSE and MoCA scores increased; and the MoCA sub-items improved in visual–spatial and executive function, language, abstraction, delayed recall, and orientation after rTMS treatment (Table 4).

**Table 4:** Comparison of relevant scale scores before and after rTMS treatment.

Characteristics	Before treatment	After treatment	*t*	*p*
MMSE	18.78 ± 4.15	25.04 ± 2.63	−5.237	<0.001^*^
Tinetti Balance	14.24 ± 1.14	14.67 ± 0.80	−4.376	<0.001^*^
Tinetti Gait	9.76 ± 1.71	10.35 ± 1.44	−7.202	<0.001^*^
BBS	46.93 ± 5.71	48.29 ± 5.13	−7.556	<0.001^*^
MoCA	19.43 ± 4.66	24.17 ± 3.81	−5.74	<0.001^*^
Visual–spatial and executive	2.49 ± 1.27	4.04 ± 0.73	−7.998	<0.001^*^
Naming	2.72 ± 0.50	2.85 ± 0.361	−1.476	0.146
Attention	4.98 ± 1.12	4.68 ± 1.25	1.218	0.229
Language	1.67 ± 0.91	2.20 ± 0.79	−3.499	0.001^*^
Abstraction	1.17 ± 0.77	1.52 ± 0.57	−2.828	0.007^*^
Delayed recall	1.48 ± 1.42	3.39 ± 1.38	−6.78	<0.001^*^
Orientation	5.11 ± 1.13	5.50 ± 0.86	−2.093	0.041^*^

^*^p < 0.05; *p*-values are the result of paired t and *χ*^2^ tests. rTMS, repetitive transcranial magnetic stimulation; MMSE, Mini-Mental State Examination; BBS, Berg Balance Scale; MoCA, Montreal Cognitive Assessment.

## 4 Discussion

In this study, wearable devices were used to evaluate objective data on multifaceted gait problems in patients with PD before and after high-frequency rTMS treatment. Statistical analysis revealed that compared with those before rTMS treatment, the mean step length, mean stride velocity, mean stride length, mean step frequency, and mean double support of patients with PD after treatment changed in the timed up and go, narrow-track, and turning tests. The gait scale was also used for evaluation, and it was found that after rTMS treatment, the scores of the Tinetti Balance Scale, Tinetti Gait Scale, and Berg Balance Scale increased. The results indicated improvements in the gait problems of patients with PD after high-frequency rTMS treatment. Abnormal excitation of the cortex and abnormal brain activity are believed to cause movement disorders in PD (Grafton, 2004; Cantello et al., 2002). Progressive loss of dopaminergic neurons is a major cause of impaired function of the cortico-basal ganglia-thalamo-cortical motor circuits in patients with PD (Braak and del Tredici, 2008). In particular, the ability of the thalamus to project to various cortical targets is easily inhibited, affecting the functional connectivity of multiple regions. Relevant studies have shown that (Chung and Mak, 2016) rTMS can improve upper limb function, walking performance, and motor signs in patients with PD in the short term. Kim et al. (2015) detected significant improvements after rTMS treatment in patients with PD with a modified standing-start 180° turn test (SS-180), freezing of gait questionnaire, timed up-and-go test, and Unified Parkinson’s Disease Rating Scale III score, and these improvements were still detectable 1 week after stimulation was discontinued. Maruo et al. (2013) treated patients with PD with high-frequency rTMS three times a day and found that these patients walked faster and had an increased stride length. High-frequency (5 Hz or higher) stimulation of the primary motor cortex has been found to significantly improve motor symptoms in patients with PD (Zanjani et al., 2015). Furthermore, rTMS improves gait characteristics by moving the leg region of the cortex and inducing alterations in cortical excitability (Yokoe et al., 2018; Lefaucheur et al., 2004). rTMS can also affect basal ganglia circuits not located at the site of stimulation, which may be a potential basis for its therapeutic effect (Strafella et al., 2003). A previous study reported that (Hamada et al., 2008) rTMS had a clinical effect on the supplementary motor area anterior to the M1 leg region, while another study (Chung et al., 2020) demonstrated that either high- or low-frequency rTMS can improve motor symptoms in patients with PD and the supplementary motor area. Lomarev et al. (2006) performed rTMS at 25 Hz for bilateral M1 and the dorsolateral prefrontal cortex (DLPFC) for 8 weeks and found that this resulted in sustained improvement in gait and bradykinesia; they subsequently hypothesized that this is the result of long-term repetitive stimulation and circuit reconstruction. There are also opposing views, Brys et al. (2016) reported that DLPFC rTMS is not better than sham. Targeting both M1 and DLPFC in each rTMS session showed no evidence of synergistic effects. Sedláčková et al. (2009) did not demonstrate any effect of high frequency rTMS applied over the DLPFC on motor performance in patients with PD. Overall, our results are similar to previous studies, but there are some differences, and studies on the long-term effects of high-frequency rTMS therapy on motor function are lacking. We still need to expand the sample size in the future.

In this study, we used the Eyeknow eye-tracking assessment system and the MoCA scale to assess the degree of cognitive function in patients with PD. Our study included three saccade tests: the prosaccade (reflective saccades), anti-saccade, and smooth pursuit tests. We found that, compared with those before rTMS treatment, the latency period of patients with PD in overlapping saccades decreased, the completion time of overlapping saccades decreased, and the average saccade speed increased after rTMS treatment. In the anti-saccade test, the completion time decreased and the average saccade speed increased. These results indicated that the cognitive function of patients with PD improved after high-frequency rTMS treatment. The cognitive demands of anti-saccade tasks have been described as inhibitory control and are related to the function of the basal ganglia and frontal regions (Li et al., 2023; Perneczky et al., 2011). Rektorová and Anderková (2017) reviewed the potential therapeutic effect of rTMS and showed that rTMS has a positive effect on cognitive impairment. Moreover, Jiang et al. (2020) tested the effect of rTMS on cognitive function in patients with PD, revealing that high-frequency rTMS of the dorsolateral prefrontal lobe may have a positive effect on executive function in these patients. There are also opposing views, Sedláčková et al. (2009) have shown that no effects of rTMS applied over the DLPFC on cognitive performance in PD patients. Pal et al. (2010) suggested that no effect could be detected over the DLPFC. Okada et al. (2021) found that the success rate of anti-saccade improved after rTMS treatment while also exhibiting faster retrosaccade latency and lower fixation saccade frequency, with no significant change in visually guided saccades. Their results have some similarities with our own. However, there are only a few studies on the effect of rTMS on eye-movement control in patients with PD. Furthermore, a 54-month prospective study by Stuart et al. (2019) showed that a smaller reflex saccade amplitude, slower mean velocity, and shorter baseline latency predicted memory loss in patients with PD. One study (Macaskill et al., 2012) has shown that the worsening of visually guided saccades correlates with the severity of cognitive decline. It has also been demonstrated (Perneczky et al., 2011) that the lateral prefrontal cortex is a crucial area for saccade control and plays a central role in executive function. Executive dysfunction is the most prominent manifestation of Parkinson’s disease cognitive dysfunction. Amador et al. (2006) showed that executive dysfunction in PD was associated with a higher rate of anti-saccade error, more inhibitions in a delayed anti-saccade task, and a longer saccade reaction time. In this study, we used the Cognitive Function Scale to assess whether the scores of the MMSE and MOCA were increased compared with those before rTMS treatment, with the results demonstrating improvements in the MOCA sub-items, visual–spatial and executive function, language, abstraction, delayed recall, and orientation. Combined with the parameters of eye-movement assessment, we believe that patients can perform eye-movement tasks better after rTMS treatment, which has a certain effect on cognitive improvement. However, we did not find any improvement in smooth pursuit after rTMS treatment. Overall, our results are similar to previous studies, but there are some differences, and a larger sample size is required for follow-up in the future.

In summary, we confirmed and assessed the effect of rTMS on motor symptoms and eye-movement performance in patients with PD. A previous study confirmed that high-frequency rTMS treatment can improve both gait and eye movement in patients with PD. Okada et al. (2021) suggested that the success rate of anti-saccade may be an indirect biomarker for assessing the effect of rTMS on gait and motor symptoms. Walton et al. (2015) also showed that patients with freezing of gait perform worse on anti-saccade tasks than those without it; this is driven by specific impairments that inhibit responses to targets in retrosaccade trials. rTMS may affect the common neural network associated with gait disorder and anti-saccade eye movements, and rTMS has therapeutic effects on both motor and non-motor symptoms in patients with PD, which may provide new directions for research on treating PD.

This study also has several limitations that warrant discussion, including the short duration of the intervention, the reproducibility of the effect of rTMS in the same patient, and the lack of sham stimulation and normal controls to elucidate the effect of rTMS on movement and cognitive impairment in patients with PD. The variable effects of the drug on cognition and motor function should also be considered. Because of the low incidence of PD and the small sample size of the study, which may affect the experimental results, larger sample sizes are required in future studies to reduce possible bias and error. In addition, we did not analyze the specific associations between cognition, eye-movement parameters, and gait disorders. The next step of this project will be to study the correlation between gait and cognitive dysfunction in depth to make breakthroughs.

In conclusion, high-frequency rTMS may be an effective therapy for improving gait disorders and cognitive functions in patients with PD. In the future, it is expected that the gait disorder and cognitive function of patients with PD will be quantitatively evaluated based on wearable devices and eye-movement assessments, allowing for changes in gait disorder and cognitive function to be dynamically monitored and evaluated using smart medical devices. Furthermore, targeted interventions can be formulated to improve the motor and non-motor symptoms in patients with PD, which may have value in guiding future work on the potential mechanisms of gait disorder and cognitive function.

## Data Availability

The original contributions presented in the study are included in the article/supplementary material, further inquiries can be directed to the corresponding authors.
